# Cannabinoid Therapy for Refractory Trigeminal Neuropathic Pain: Quantification of Somatosensory Alterations (QualST) by 3D Stereophotogrammetry—Two Case Reports

**DOI:** 10.1155/crid/7752444

**Published:** 2026-05-04

**Authors:** Alex Moreira Mélo, Luiz Guilherme Spadon-Brito, Júia Carrer Hallak, Glauce Crivelaro do Nascimento, Melissa de Oliveira Melchior, Simone Cecílio Hallak Regalo, Jardel Francisco Mazzi-Chaves, Laís Valencise Magri

**Affiliations:** ^1^ Department of Restorative Dentistry, School of Dentistry of Ribeirão Preto, University of São Paulo (FORP/USP), Ribeirão Preto, SP, Brazil, usp.br; ^2^ Department of Basic and Oral Biology, School of Dentistry of Ribeirão Preto, University of São Paulo (FORP/USP), Ribeirão Preto, SP, Brazil, usp.br

**Keywords:** cannabinoids, case report, medical cannabis, orofacial pain, trigeminal neuralgia

## Abstract

**Background:**

Trigeminal neuropathic pain (TNP) is a chronic and debilitating condition frequently resistant to conventional pharmacological therapies. Although cannabinoids have emerged as a potential adjunctive treatment, objective clinical documentation of their effects in orofacial neuropathic pain remains limited.

**Case Reports:**

We report two female patients with refractory TNP—one post‐traumatic and one idiopathic—who experienced insufficient relief despite trials of anticonvulsants, antidepressants, topical agents, and local interventions. Both patients received a balanced tetrahydrocannabinol (THC):cannabidiol (CBD) sublingual formulation (20 mg/mL each) following a structured titration protocol and were monitored over 8 weeks. Outcomes were assessed using the Douleur Neuropathique 4 Questions (DN4), the visual analog scale (VAS), the World Health Organization Quality of Life‐BREF (WHOQOL‐Bref), and qualitative sensory testing (QualST). Additionally, three‐dimensional facial stereophotogrammetry was employed to objectively quantify areas of hyperfunction and allodynia. Treatment resulted in a marked analgesic response (VAS reduction from 9 to 4 and from 10 to 2), qualitative changes in pain phenotype—from shock‐like to predominantly burning—and functional improvement in mastication and oral hygiene. Stereophotogrammetry demonstrated a substantial reduction in sensitized regions, with extraoral hyperfunctional area decreasing from 113.72 to 27.54 cm^2^ and complete resolution of allodynia by week 8. WHOQOL‐Bref scores improved in both patients, with physical domain scores increasing from 50.0 to 60.7 and from 25.0 to 39.3, accompanied by gains in psychological well‐being. No serious adverse events were recorded.

**Conclusion:**

These cases illustrate the potential clinical relevance of cannabinoids as an adjunctive approach for refractory TNP and highlight the importance of multidimensional assessment strategies. The findings should be interpreted cautiously given the descriptive nature of case reports; however, they underscore the need for controlled studies to further investigate the efficacy and safety of cannabinoid‐based therapies in orofacial neuropathic pain.

## 1. Introduction

Neuropathic pain is defined by the International Association for the Study of Pain (IASP) as pain caused by a lesion or disease directly affecting the somatosensory system [1, 2]. It is a complex and multifactorial condition involving peripheral and central mechanisms, as well as genetic, inflammatory, and immunological influences. Clinically, it is characterized by symptoms such as burning, electric shocks, tingling, hyperalgesia, and allodynia, often associated with emotional repercussions, including anxiety and depression, which may intensify suffering and contribute to pain chronification [3, 4].

In the field of orofacial pain, painful trigeminal neuropathy (PTN) stands out as a condition resulting from injury or dysfunction of the trigeminal nerve or its peripheral branches. Unlike classical trigeminal neuralgia, which is characterized by paroxysmal shock‐like attacks, PTN typically presents as continuous or persistent pain accompanied by objective somatosensory changes such as hypesthesia, hyperalgesia, and allodynia [5, 6]. This conceptual distinction represented an important milestone for clinical practice and supported the development of the International Classification of Orofacial Pain [7], which categorizes trigeminal neuropathic pain (TNP) into three main groups: idiopathic TNP, post‐traumatic TNP (PTTNP), and neuropathic pain attributed to herpes zoster.

PTTNP is particularly relevant in dentistry, as it may occur after invasive procedures such as extractions, implant placement, orthognathic surgery, and endodontic interventions. Its prevalence is estimated at 3%–5% following injuries to peripheral branches of the trigeminal nerve, especially after third molar extractions and dental implant placement [8]. Idiopathic TNP, in contrast, remains less understood due to the absence of identifiable trauma or specific etiology. Nevertheless, affected patients present persistent neuropathic pain, suggesting multifactorial predisposition or central sensitization mechanisms that are not yet fully elucidated [3].

Both forms pose significant clinical challenges. Conventional management typically includes anticonvulsants (such as carbamazepine and gabapentinoids), tricyclic antidepressants, serotonin–norepinephrine reuptake inhibitors, local anesthetic blocks, and, in refractory cases, neurosurgical interventions [9]. However, these therapies frequently provide incomplete pain relief and may be associated with clinically relevant adverse effects. Carbamazepine, considered a first‐line treatment, has been linked to dizziness, somnolence, and hematological reactions that may lead to treatment discontinuation, while antidepressants and gabapentinoids can produce sedation, cognitive impairment, and gastrointestinal symptoms, potentially limiting long‐term adherence. These therapeutic challenges have encouraged the investigation of safer and more tolerable alternatives.

In this context, medical cannabis has emerged as a promising therapeutic approach. *Cannabis sativa* contains more than 100 phytocannabinoids, among which delta‐9‐tetrahydrocannabinol (THC) and cannabidiol (CBD) are the most extensively studied. These compounds interact with the human endocannabinoid system, which plays a central role in modulating pain perception, inflammation, and neuroprotective processes [10]. THC exhibits recognized analgesic properties, although it is associated with psychoactive effects [11], whereas CBD is nonpsychotropic and demonstrates anti‐inflammatory, antioxidant, anxiolytic, and anticonvulsant actions [9, 12, 13]. Evidence suggests that the combination of both compounds may exert synergistic effects, enhancing analgesia while improving tolerability [13].

Recent clinical studies indicate that cannabinoids may reduce peripheral and central neuronal hypersensitivity by modulating CB1 and CB2 receptors and attenuating inflammatory responses, thereby conferring neuroprotective effects [14, 15]. Observational trials involving patients with trigeminal neuralgia have also reported significant pain reduction and decreased opioid consumption following treatment with combined THC/CBD formulations [16].

In Brazil, since the publication of RDC 660/2022 by Anvisa, the prescription of cannabis‐derived products for medical purposes has been regulated, and dentists have been recognized by the Federal Council of Dentistry as qualified professionals for such practice. Additionally, the establishment of the Brazilian Society of Cannabinoid Dentistry has reinforced the importance of scientific dissemination and professional training in this emerging field.

Clinical case reports play a fundamental role in advancing knowledge in emerging therapeutic areas by providing preliminary evidence on efficacy and safety, illustrating real‐world applications, and introducing innovative methods for monitoring therapeutic responses. The present report aims to contribute to this field by describing two patients with refractory TNP—one with post‐traumatic etiology and the other idiopathic—treated with a THC:CBD sublingual extract as an adjunct to ongoing conventional pharmacological management, which remained stable throughout the observation period. Beyond conventional clinical and psychometric assessments, three‐dimensional stereophotogrammetry was employed to objectively quantify changes in sensitized facial areas, offering a novel methodological contribution to the evaluation of therapeutic outcomes in orofacial pain.

## 2. Methodology

The clinical cases were approved by the Research Ethics Committee of the School of Dentistry of Ribeirão Preto (FORP/USP), under protocol CAAE: 92507925.7.0000.5419. The patients were fully informed about the study procedures and voluntarily signed the informed consent form prior to participation.

Therefore, the present study describes two clinical cases of refractory TNP, one post‐traumatic and the other idiopathic, in which medical cannabis was proposed as part of pain management.

**Figure 1:**
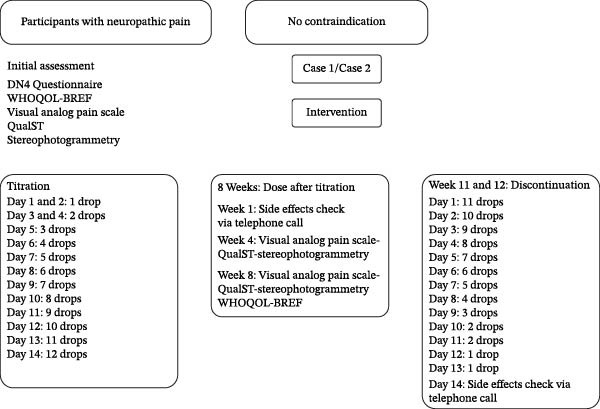
Schematic representation of the therapeutic protocol for cannabinoid intervention, comprising four sequential phases: baseline (1 week), titration (2 weeks), stable‐dose phase (8 weeks), and discontinuation (2 weeks). Clinical evaluations (QualST, VAS, DN4), quality‐of‐life assessments (WHOQOL‐Bref), and safety monitoring were performed at predefined intervals.

The medication was provided by the Associação Brasileira de Acesso do Rio de Janeiro (ABRARIO), a nonprofit civil entity (CNPJ n° 40.519.119/0001‐90), headquartered in Niterói (RJ). ABRARIO is legally authorized to carry out its activities, according to the valid license from the competent health authority, being responsible for obtaining the active pharmaceutical ingredient (API) and preparing the medication in compliance with Brazilian legislation on medical cannabis. A chronological timeline summarizing symptom onset, prior interventions, cannabinoid initiation, dose stabilization, and follow‐up assessments is presented in Figure 1, where the cases are organized into three phases: initial evaluation (baseline), intervention (titration and stable dose), and discontinuation.

### 2.1. Formulation

The cannabinoid formulation used in this study consisted of a balanced full‐spectrum extract containing Δ9‐THC and CBD in a 1:1 ratio. The product presented a concentration of 40 mg/mL (20 mg/mL THC and 20 mg/mL CBD) in a 30‐mL bottle, with medium‐chain triglycerides (MCT) as the excipient vehicle. Each milliliter corresponded to ~32 drops. Based on this proportion, each drop delivered ~100 µL, equivalent to 2.7 mg of cannabinoids (2.5 mg of THC and 2.5 mg of CBD of effective content). The chemical identity of CBD is 2‐[(1R,6R)‐3‐methyl‐6‐(1‐methylethenyl)‐2‐cyclohexen‐1‐yl]‐5‐pentyl‐1,3‐benzenediol (CAS: 13956‐29‐1; molecular formula C_21_H_30_O_2_), and THC corresponds to (6aR,10aR)‐6,6,9‐trimethyl‐3‐pentyl‐6a,7,8,10a‐tetrahydro‐6H‐benzo[c]chromen‐1‐ol. The formulation was manufactured under pharmaceutical‐grade standards, and dosing was administered sublingually. Titration was individualized according to tolerability and clinical response, with a maximum daily limit of 12 drops (~30 mg of combined cannabinoids), in accordance with current regulatory guidelines for medical cannabis use.

The choice of a balanced 1:1 THC:CBD formulation was based on evidence suggesting potential synergistic analgesic effects between THC and CBD, with improved tolerability compared to high‐THC formulations. THC exerts central analgesic effects through CB1 receptor modulation, whereas CBD contributes anti‐inflammatory and neuromodulatory actions without psychoactive properties, potentially mitigating adverse effects associated with THC. A gradual titration protocol was adopted in accordance with clinical recommendations for medical cannabis in neuropathic pain, aiming to optimize analgesia while minimizing dose‐related adverse effects. The maximum daily limit was established based on regulatory guidance and published clinical experience in cannabinoid‐based therapies for chronic neuropathic pain.

The cannabinoid extract was produced according to a standardized internal Standard Operating Procedure (SOP; production code LAB06), ensuring preparation consistency and traceability. Two batches of cannabis oil were used in the present study (EBPK0124 and EBSN0525). Batch EBPK0124 underwent physicochemical and microbiological quality‐control testing performed by an external accredited laboratory (DALL PhytoLab, Curitiba, Brazil), including cannabinoid quantification by high‐performance liquid chromatography (HPLC; POP.401), residual solvent analysis by gas chromatography with headspace detection (GC‐Headspace; POP.326), and microbiological testing according to Farmacopeia Brasileira VII standards. Batch EBSN0525 underwent independent phytocannabinoid profiling at the Toxicology Analytical Laboratory, Campinas Poison Control and Toxicological Assistance Center (CIATox‐Campinas), University of Campinas (UNICAMP), Brazil, using HPLC‐DAD methodology (POP LTA MET‐402) for full cannabinoid panel quantification. Batch identification and analytical documentation ensured traceability, product standardization, and interbatch consistency throughout the study period.

### 2.2. Initial Evaluation

Before titration, all patients underwent neuropathic pain screening using the Douleur Neuropathique 4 Questions (DN4), health‐related quality‐of‐life assessment using the World Health Organization Quality of Life‐BREF (WHOQOL‐Bref), pain intensity assessment using the visual analog scale (VAS), and somatosensory mapping using qualitative sensory testing (QualST). In addition, three‐dimensional facial stereophotogrammetry was performed to quantify extraoral areas of hyperfunction and allodynia.

### 2.3. Intervention

The intervention was structured in four stages. During the baseline period (1 week), initial symptoms and clinical parameters were recorded, without pharmacological intervention. Subsequently, the titration phase (2 weeks) began, in which patients followed a progressive escalation protocol: days 1–2 with 1 drop/day, days 3–4 with 2 drops/day, day 5 with 3 drops/day, day 6 with 4 drops/day, day 7 with 5 drops/day, until reaching 12 drops/day on day 14, according to clinical tolerance. The maximum limit was set at 12 drops within 24 h, and self‐titration was guided by clinical efficacy and the presence of adverse effects, with instructions to stop dose escalation once symptom stabilization occurred.

After titration, the stable dose phase (8 weeks) was established, during which patients maintained the final dose for eight consecutive weeks. In this period, a telephone consultation was conducted in week 1 to monitor adverse effects, an in‐person reassessment in week 4 with application of QualST, VAS, and facial stereophotogrammetry (when indicated), and a final evaluation in week 8 with QualST, VAS, stereophotogrammetry, and WHOQOL‐Bref. Throughout this phase, other pain medications were maintained at stable doses, with acetaminophen allowed as rescue medication, in addition to ongoing concomitant therapies (occupational therapy and use of a stabilization interocclusal device). Finally, the discontinuation phase (2 weeks) consisted of the gradual withdrawal of the drug in reverse titration scheme until complete suspension. A follow‐up phone call was conducted 2 weeks after discontinuation to assess possible withdrawal symptoms, clinical stability, and adverse effects (Figure 1). Adverse events were actively and systematically assessed at each scheduled contact, rather than based solely on spontaneous reporting.

### 2.4. Assessment Instruments

**Figure 2:**
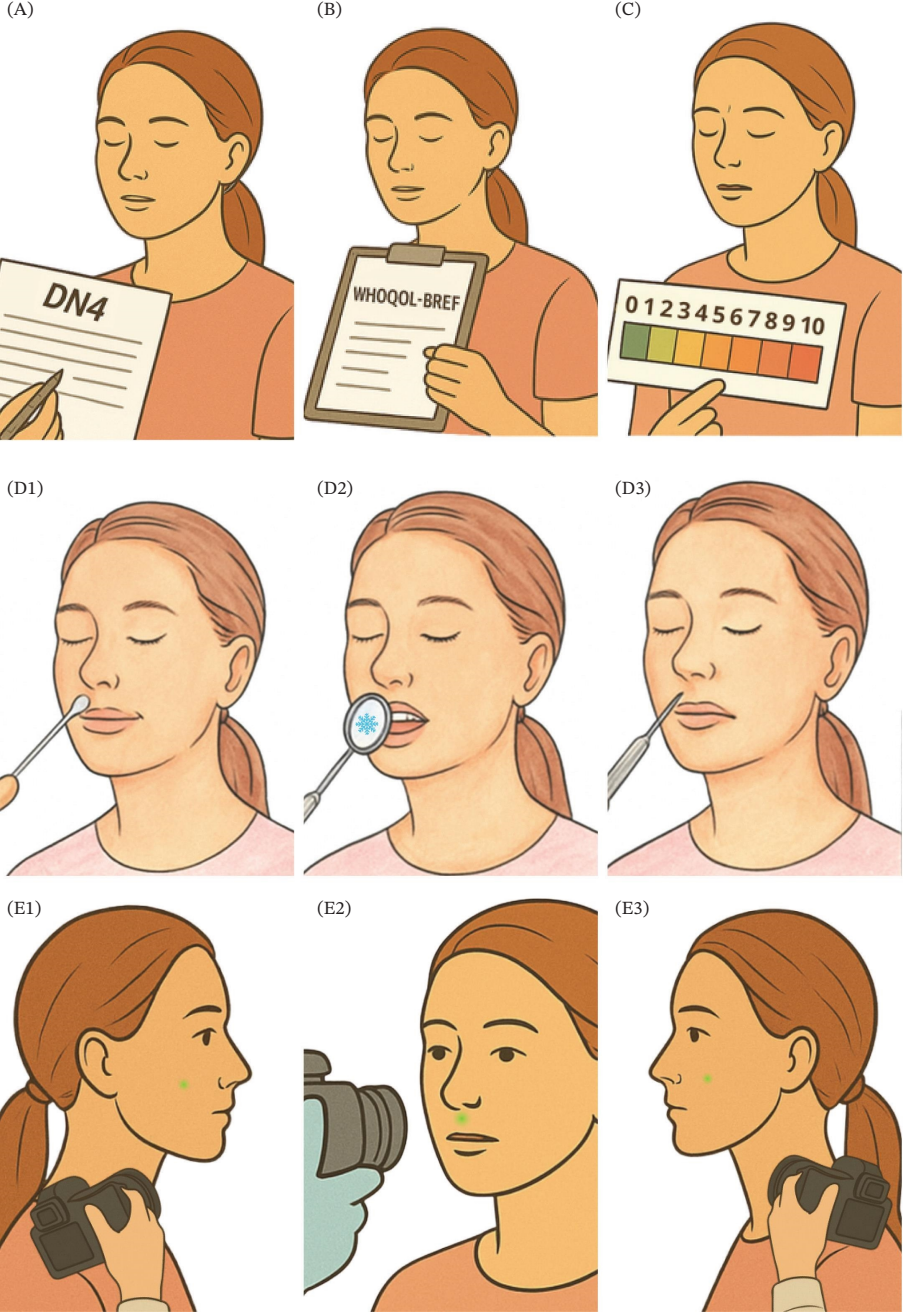
Overview of the multimodal assessment protocol applied in both clinical cases. Instruments included: (A) Douleur Neuropathique 4 Questions (DN4) for neuropathic pain screening; (B) WHOQOL‐Bref for health‐related quality of life; (C) visual analog scale (VAS) for pain intensity; (D) qualitative sensory testing (QualST) for intraoral somatosensory mapping (light touch, cold, and pinprick); and (E) three‐dimensional facial stereophotogrammetry for objective quantification of extraoral sensitization and hyperfunction areas.

Standardized and validated methods were employed to characterize and quantify neuropathic pain, as illustrated in Figure 2.

Neuropathic pain screening was conducted using the DN4 Questions instrument, which consists of seven patient‐reported symptom items and three clinician‐observed sensory signs. Light touch was assessed using a sterile cotton swab and pinprick sensation with a steel dental probe. Each positive item scored one point, yielding a total score from 0 to 10, with values ≥4 indicative of neuropathic pain.

Health‐related quality of life was evaluated using the WHOQOL‐Bref, a 26‐item questionnaire encompassing four domains: physical, psychological, social, and environmental. Items were scored on a five‐point Likert scale and subsequently transformed to a 0–100 metric, where higher scores reflect better perceived quality of life. The instrument was self‐administered in a quiet setting and required ~15 min to complete.

Pain intensity was assessed using a VAS, consisting of a 10‐cm horizontal line anchored at “0” (no pain) and “10” (worst imaginable pain). Participants were instructed to indicate the point corresponding to their current pain level.

Somatosensory function was examined through QualST. Stimuli included light touch (sterile cotton swab), cold sensation (dental mirror cooled with cold spray), and pinprick (sterile dental probe). Each stimulus was applied in random order to both the symptomatic trigeminal territory and its contralateral counterpart. Participants classified sensation as normal, decreased (hypofunction), or increased (hyperfunction).

Additionally, three‐dimensional facial stereophotogrammetry was performed using the Vectra H1 portable imaging system (Canfield Scientific). Imaging was conducted under controlled lighting conditions with patients seated in a neutral position. Standardized frontal and bilateral profile photographs were acquired using anatomically referenced positioning. The Vectra H1 utilizes dual stereoscopic cameras and structured‐light projection to generate high‐resolution 3D surface models through triangulation, achieving sub‐millimetric accuracy (~0.2 mm). Image alignment was performed in Vectra Analysis Module software using anatomical landmarks (tragus, lateral canthus, nasion, pogonion). Clinically identified regions of hyperfunction were digitally demarcated, and surface area (cm^2^) was computed for quantitative monitoring of treatment‐related changes. Three‐dimensional stereophotogrammetry is commonly employed in longitudinal craniofacial assessments for accurate measurement of surface area and volumetric changes, demonstrating high reproducibility and spatial precision. In the present study, this technique was applied as a standardized and objective tool for documentation and quantitative evaluation of extraoral somatosensory alterations over time.

### 2.5. Case 1: PTTNP

A female patient, 45 years old, presented to the Stomatology Service of the Ribeirão Preto School of Dentistry (FORP/USP) with complaints of persistent pain in the apical region of tooth 16 radiating toward the edentulous area of tooth 17. The pain had begun 7 months earlier, following an unsuccessful endodontic treatment on tooth 17, subsequent extraction, and maxillary sinus lift performed for future implant placement. Her medical history was significant for depression and fibromyalgia. She was under continuous pharmacotherapy with carbamazepine (400 mg/day, 200 mg twice daily), sertraline (50 mg/day), and amitriptyline (50 mg/day).

**Figure 3:**
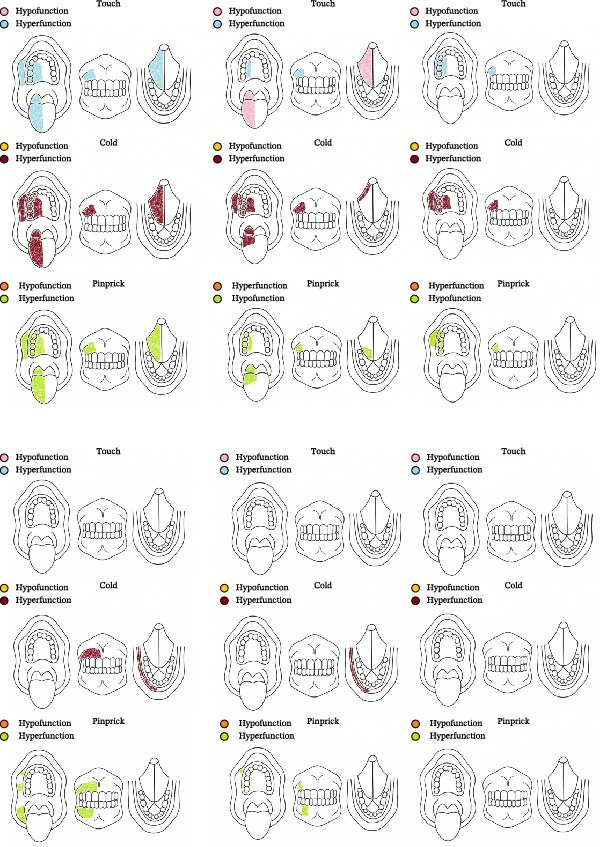
Intraoral Qualitative Sensory Testing (QualST) heatmaps depicting sensory responses across three modalities—mechanical allodynia, cold hyperalgesia, and pinprick hyperfunction—for Case 1 (upper panels) and Case 2 (lower panels). Assessments were performed at baseline (week 0), week 4, and week 8 of cannabinoid treatment.

At the initial evaluation, she described the pain as severe, burning, and tingling, accompanied by numbness and recurrent paresthesia in the affected region, scoring 9 on the VAS. Baseline assessments included the DN4, WHOQOL‐Bref, VAS, and QualST. DN4 revealed burning pain, tingling, numbness, and pain elicited by light tactile stimulation, consistent with a neuropathic profile. QualST demonstrated sensory hyperfunction to touch, cold, and pinprick stimuli in teeth 15–17, as well as in the dorsal and ventral surfaces of the tongue and the palate (Figure 3). WHOQOL‐Bref scores were physical = 50.0, psychological = 50.0, social relationships = 50.0, and environment = 40.6, with an aggregate score of 190.6.

The diagnosis of PTTNP was established according to the ICOP (2020). The patient presented with persistent unilateral facial pain localized to the maxillary branch (V2) following a documented peripheral nerve injury during dental procedures, with symptom onset within 6 months of trauma. The pain was characterized by burning and tingling sensations, associated with paresthesia and mechanical allodynia, and accompanied by somatosensory abnormalities confirmed by clinical examination and QualST. Radiographic and clinical evaluations excluded odontogenic or other secondary causes, fulfilling ICOP diagnostic criteria for PTTNP.

Multiple prior therapeutic strategies had failed to provide significant relief. The patient had previously been treated with pregabalin (150 mg/day), dexamethasone (4 mg/day), dipyrone (500 mg every 6 h as needed), topical lidocaine (Toperma 5%), capsaicin 0.08% combined with 2% lidocaine, and topical amitriptyline 5%. Local anesthetic nerve blocks and surgical exploration of the trigger zone were also attempted without sustained benefit. A neurologist‐administered anesthetic block provided only transient pain reduction.

Due to therapeutic refractoriness, a structured titration protocol with medical cannabis was initiated. The formulation consisted of a 1:1 THC:CBD sublingual extract (20 mg/mL each; Abrario). The patient began with 1 drop/day, progressively increasing to 10 drops/day, divided into two daily doses. During the initial titration period, mild adverse effects were reported—dizziness, drowsiness, and increased appetite—which were successfully managed by adjusting the dosage to 4 drops in the morning and 6 drops at night. Analgesic response, adverse events, and potential drug interactions were systematically recorded throughout the treatment.

Follow‐up evaluations were conducted at weeks 4 and 8 for DN4, VAS, and QualST, while the WHOQOL‐Bref was applied again at week 8. VAS scores showed progressive improvement, decreasing from 9 (baseline) to 8 (week 4), 6 (interim week 8), and 4 (final week 8) (Graph 1). QualST revealed a gradual reduction in hyperfunction to tactile, thermal, and pinprick stimuli. Initially widespread across the tongue, palate, buccal mucosa, and teeth 14–18, the sensory hyperfunction became restricted after 2 months to the palatal and vestibular regions of teeth 14–17, with marked reduction on the tongue (Figure 3).

At week 8, WHOQOL‐Bref scores improved to: physical = 60.7, psychological = 58.3, social relationships = 58.3, and environment = 46.9, yielding an aggregate score of 224.2. These results reflected a transition from moderate to good quality of life, particularly in the physical and psychological domains (Table 1).

**Table 1:** WHOQOL‐Bref scores in Cases 1 and 2, assessing physical, psychological, social, and environmental domains before and after cannabinoid treatment.

Domain	Case 1 before	Case 1 after	Case 2 before	Case 2 after
Physical	50.0	60.7	25.0	39.3
Psychological	50.0	58.3	70.8	66.7
Social	50.0	58.3	66.7	75.0
Environment	40.6	46.9	56.3	53.1
Total aggregate	190.6	224.2	218.8	234.1

*Note*: Results show improvements across all domains.

After completion of the stable‐dose phase, the cannabinoid formulation was gradually tapered, decreasing by 25 mg every 5 days until complete discontinuation. No withdrawal symptoms were observed. The patient maintained stable analgesia and quality‐of‐life improvements, indicating a clinically meaningful improvement and acceptable tolerability during the monitored period, without allowing causal inferences.

**Figure 4:**
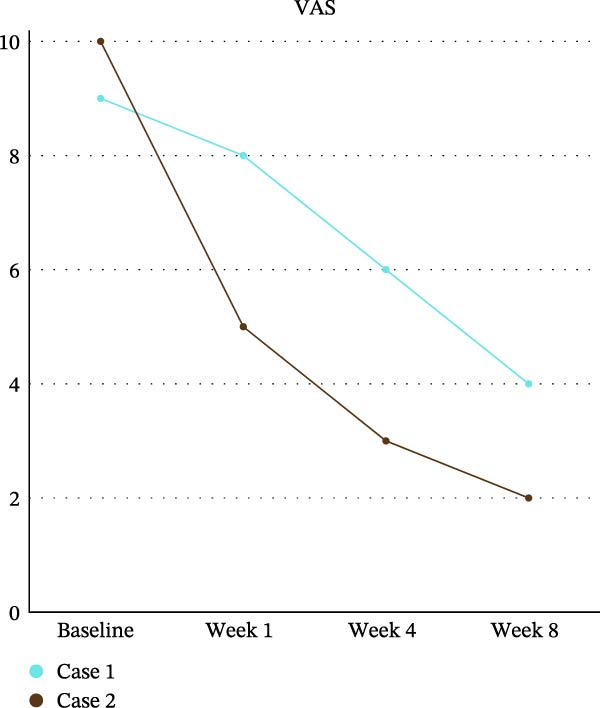
Longitudinal trajectory of pain intensity (VAS) in both cases from baseline through week 8. Both patients exhibited clinically meaningful reductions in pain scores over time, consistent with improved somatosensory function and quality‐of‐life outcomes.

A marked reduction in pain intensity was observed over time, with a progressive decrease in VAS scores from baseline to week 8. The most pronounced improvement occurred within the first weeks, followed by a continued but more gradual decline, indicating a sustained therapeutic effect. As illustrated in Figure 4, pain levels approached minimal values by the end of the follow‐up period.

### 2.6. Case 2: Idiopathic TNP With Concomitant Continuous Pain

A female patient, 50 years old, sought care at the Orofacial Pain and Temporomandibular Disorders Service of the Ribeirão Preto School of Dentistry (FORP/USP) complaining of persistent shock‐like pain for over 5 years, affecting intraoral and extraoral regions corresponding to V2 and V3 territories. Pain was localized to the zygomatic bone region (V2) and extended from the labial commissure to the mandibular angle (V3). No causal event was identified. Her medical history included diabetes and hypertension. She was on continuous use of carbamazepine (800 and 400 mg twice daily), metformin (850 mg), propranolol (40 mg), losartan (25 mg), and hydrochlorothiazide (25 mg). At initial evaluation, she reported severe pain described as shocks, tingling, and pinprick sensations, with recurrent paresthesia in the affected region, scoring 10 on VAS.

**Figure 5:**
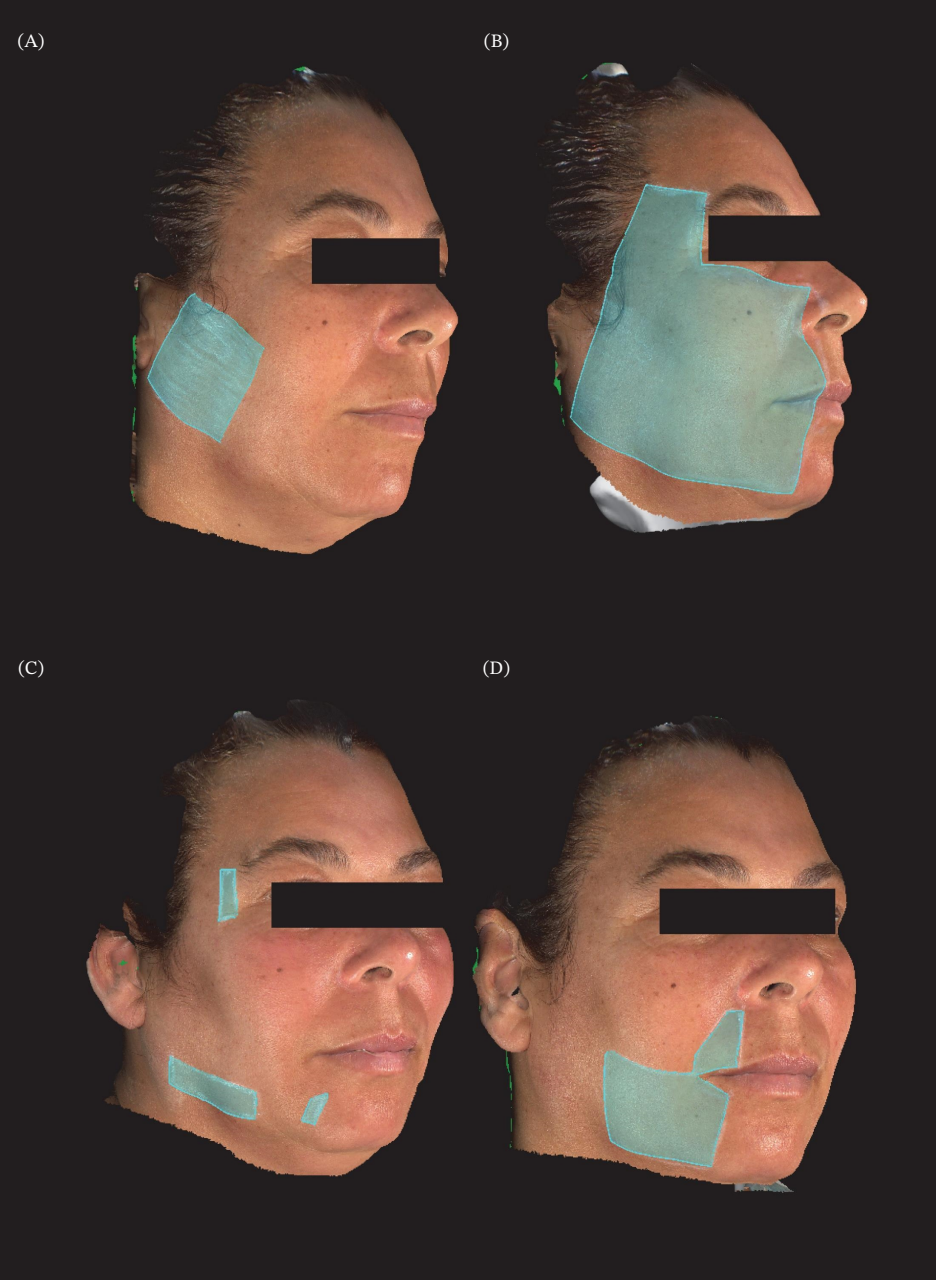
Extraoral somatosensory evaluation with QualST and 3D facial stereophotogrammetry in Case 2. (A) Baseline map of mechanical allodynia; (B) baseline map of pinprick hyperfunction; (C) pinprick hyperfunction at week 4; (D) pinprick hyperfunction at week 8.

Initial assessments included the same assessments described in Case 1. DN4 revealed shock‐like pain, tingling, pinpricks, and pain triggered by light friction, consistent with neuropathic pain. QualST showed hyperfunction to pinprick stimuli in teeth 11–17, 42–47, and right buccal mucosa; to cold stimuli on the right lower lip and teeth 11–17, without intraoral allodynia (Figure 3). Extraorally, hyperfunction to pinprick was observed in V1, V2, and V3 territories and to touch in V2 and V3 (Figure 5). WHOQOL‐Bref scores were physical = 25.0, psychological = 70.8, social relationships = 66.7, and environment = 56.3, with an aggregate score of 218.8.

The diagnosis of idiopathic TNP with concomitant continuous pain was made according to ICOP (2020) criteria. The patient reported persistent unilateral facial pain in the maxillary and mandibular divisions (V2 and V3) for over 3 months, characterized by shock‐like and burning sensations, without evidence of trauma, dental pathology, or neurological disease capable of explaining the symptoms. Somatosensory dysfunction was confirmed through QualST, demonstrating hyperalgesia and allodynia in the affected trigeminal distributions. The absence of identifiable structural or traumatic etiology, combined with consistent clinical and sensory findings, satisfied ICOP diagnostic criteria for idiopathic TNP with concomitant continuous pain.

Treatment with medical cannabis followed the titration protocol. The first formulation was a 1:1 THC:CBD extract (20 mg/mL each—Abrario), starting with 1 drop/day and increasing up to 12 drops/day, divided into two doses. During titration, the patient reported only drowsiness, which was not considered limiting, and dosing was maintained at 6 drops in the morning and 6 drops at night.

By week 4, QualST showed reduced hyperfunction: thermal response remained only on the lower lip, with no buccal mucosa hyperfunction. By week 8, no intraoral hyperfunction remained. Vectra stereophotogrammetry quantified extraoral areas: initial allodynia, 24.42 cm^2^, hyperfunction to pinprick, 113.72 cm^2^, reduced to 11.83 cm^2^ by week 4, and to 27.54 cm^2^ at week 8. Allodynia was reduced to 0 cm^2^ at week 8.

At the first follow‐up after starting cannabinoid treatment, the patient reported excessive drowsiness as the only side effect but described a change in pain characteristics, shifting from shock‐like pain to burning pain. By week 4, a significant reduction in hyperfunction areas was observed: thermal response persisted only on the lower lip, with no hyperfunction in the buccal mucosa; the hyperfunction area to pinprick decreased to 11.83 cm^2^. At week 8, no intraoral hyperfunction was detected by QualST, while extraoral hyperfunction remained at 27.54 cm^2^, and the allodynia area was reduced to 0 cm^2^.

Clinically, at week 8, the patient reported being able to move her jaw without shock sensations, previously triggered when opening the mouth, especially at the labial commissure. She also noted improvements in daily activities such as toothbrushing and eating. After discontinuing cannabis, she remained pain‐free for 15 days, later experiencing only sporadic shocks localized to a ~ 1 cm^2^ area in the upper lip, as described (Figure 5). The WHOQOL‐Bref scores at the end of the eighth week showed improvements in the physical (60.7), psychological (58.3), social relationships (58.3), and environmental (46.9) domains, with an aggregate score of 224.2, indicating an overall perception of moderate‐to‐good quality of life, especially in the physical and psychological aspects (Table 1).

After the stable‐dose phase, the formulation was gradually tapered (25 mg reduction every 5 days) until complete discontinuation. No serious adverse effects were reported during the observation period, while improvements in pain and quality of life were documented.

Similarly, Case 2 demonstrated a consistent reduction in pain intensity across the evaluation period, although with a slightly more gradual trajectory compared to Case 1. VAS scores decreased steadily from baseline through week 8, reflecting a continuous improvement in symptoms. This pattern is clearly depicted in Figure 4, highlighting the overall effectiveness of the intervention in reducing pain.

## 3. Discussion

The present report described two patients with refractory TNP, one post‐traumatic and one idiopathic, who showed observable clinical improvement following adjunctive treatment with a balanced THC:CBD formulation. Both cases demonstrated reductions in pain intensity, qualitative changes in pain profile from paroxysmal shock‐like episodes to more tolerable continuous burning pain, and clinically relevant functional recovery in activities such as chewing, mandibular movements, and oral hygiene. These clinical observations were accompanied by improvements in quality‐of‐life domains. No serious or treatment‐limiting adverse events were identified during prospective safety monitoring, which included structured telephone follow‐up and in‐person evaluations. Importantly, the integration of three‐dimensional stereophotogrammetry provided objective documentation of the reduction of hyperfunction and allodynia areas, reinforcing the reliability of the observed outcomes and highlighting the potential of this technology as an innovative tool for monitoring therapeutic responses in orofacial neuropathic pain.

In both cases, diagnostic classification was established according to the International Classification of Orofacial Pain (ICOP, 2020), which defines TNP as pain arising from a lesion or disease affecting the trigeminal somatosensory system and characterized by persistent pain and associated sensory abnormalities. The clinical presentation differed from classical trigeminal neuralgia, which under ICOP is defined by recurrent, brief, electric shock‐like paroxysms typically triggered by innocuous stimuli and frequently associated with neurovascular compression. In the present cases, patients exhibited continuous background pain, neuropathic descriptors, and somatosensory alterations consistent with TNP rather than paroxysmal neuralgia. Neuroimaging did not reveal structural lesions, demyelinating disease, or compressive pathology. In the idiopathic case, the absence of identifiable etiological factors combined with persistent neuropathic features and positive screening for neuropathic pain supported classification within the ICOP framework as TNP rather than classical trigeminal neuralgia or secondary neuralgia.

TNP is recognized as a condition that is complex and difficult to manage. It significantly affects patients’ psychological, physical, and social behaviors [15, 16] and is often refractory to conventional pharmacological therapies [17, 18]. In the two cases reported here, different treatment modalities, including anticonvulsants and local blocks, failed to achieve satisfactory control, highlighting the need for complementary approaches in patients who do not find relief with traditional approaches.

The use of cannabinoids in both cases of refractory TNP was associated with clinically relevant changes in pain patterns and functional impact, with patients reporting a reduction in shock‐like crises replaced by burning pain that was more tolerable and less disabling. In both patients, treatment consisted of a balanced THC:CBD sublingual formulation (20 mg/mL each), titrated individually up to 10–12 drops per day and maintained for 8 weeks, while conventional pharmacological therapy remained stable throughout the observation period. This clinical context is important when interpreting the observed responses. The selection of a balanced formulation and individualized titration was guided by pharmacological evidence supporting combined cannabinoid modulation of central and peripheral pain pathways, as well as by safety considerations described in previous clinical reports [19]. Pain intensity decreased from NRS 9 to 4 in Case 1 and from NRS 10 to 2 in Case 2, accompanied by functional improvements in daily activities such as chewing, mandibular opening, and oral hygiene. Similar findings have been described in other reports, including a 46‐year‐old patient with trigeminal neuralgia whose pain decreased from NRS 8 to NRS 5 within 15 days and to NRS 3 after 90 days of cannabinoid use [20] However, available evidence remains limited and heterogeneous.

According to IMMPACT recommendations for chronic pain clinical trials, reductions of ~30% in pain intensity are generally considered clinically meaningful, whereas reductions of 50% or greater represent substantial improvement. In the present cases, pain intensity decreased by 55.6% in Case 1 and 80% in Case 2, exceeding thresholds typically associated with substantial clinical response. Although these benchmarks derive from controlled trial methodology, they provide a structured framework for contextualizing the magnitude of change observed in this descriptive report [21]. Another case involving a 54‐year‐old man with multiple sclerosis–related trigeminal neuralgia refractory to conventional treatment showed elimination of painful episodes (NRS 0/10) after 30 days of nabiximol (CBD 25 mg/mL/THC 27 mg/mL), with sustained benefits for 6 months and only mild adverse effects [17].

A recent narrative review synthesizing randomized clinical trials published between 2014 and 2024 further contextualizes these findings within the broader neuropathic pain literature [22]. Across heterogeneous neuropathic conditions, cannabinoid‐based therapies demonstrated modest reductions in pain intensity and improvements in selected quality‐of‐life measures, although results varied substantially according to formulation, route of administration, and patient population. Combined THC:CBD formulations were highlighted as potentially advantageous due to synergistic analgesic effects and mitigation of THC‐related psychoactive adverse events. Importantly, the review emphasizes persistent methodological limitations, including small sample sizes, heterogeneous diagnostic categories, and reliance on subjective outcomes, as well as the scarcity of data specifically addressing TNP. These considerations reinforce the need for cautious interpretation of the present cases and underscore the importance of controlled studies targeting well‐defined trigeminal pain phenotypes.

TNP not only causes pain but also interferes with fundamental psychological, physical, and social behaviors. It may directly affect mandibular movements and even light facial contact, explaining its considerable impact on daily functions such as chewing, kissing, toothbrushing, and shaving [18, 23]. In both cases reported here, the observed functional improvement aligns with this perspective, suggesting that cannabinoids may contribute to benefits beyond analgesia, extending to autonomy and self‐esteem.

From a physiological standpoint, the analgesic mechanisms of cannabinoids are complex and multifactorial. At the central level, activation of CB1 receptors reduces the release of excitatory neurotransmitters and neuronal excitability, diminishing the paroxysmal discharges that characterize shock‐like pain. This action involves modulation of the descending pain pathway, including the periaqueductal gray, rostral ventromedial medulla, and spinal cord [24, 25]. CBD exerts antinociceptive effects by interacting with CB1R and modulating N‐type calcium channels, 5‐HT_3_ receptor‐mediated ion channels, and GABAergic systems implicated in trigeminal neuralgia [22, 26]. Preclinical studies support that both CBD and synthetic cannabinoids inhibit hyperalgesia and allodynia through these mechanisms [18, 25]

At the peripheral level, CB2 receptors regulate inflammation and immune responses, reducing processes that perpetuate peripheral sensitization. Cannabinoid formulations have shown promise in managing chronic inflammatory conditions associated with orofacial dysfunction [15, 27]. The endocannabinoid system has also been linked to pain control in rheumatic diseases [28]. Furthermore, CBD interacts with noncannabinoid receptors such as TRPV channels. Recent studies indicate that CBD binds preferentially to TRPV2, modulating hyperalgesia and reducing pain sensitivity [29, 30]. This combination of mechanisms may help explain the alteration in pain pattern observed in the present cases, in which shock‐like pain was replaced by a more tolerable continuous pain, potentially reflecting modulation of peripheral and central sensitization [16, 31]. This combined modulation of central and peripheral mechanisms may contribute to the observed shift in pain phenotype, although mechanistic inferences remain speculative in a case‐report design.

Quality of life was systematically evaluated with WHOQOL‐Bref, assessing physical, psychological, social, and environmental domains. In both cases, cannabinoid therapy was associated with improvements across domains, reflecting benefits that extended beyond symptom control to greater autonomy and satisfaction with daily activities. This transformation of pain from disabling paroxysmal crises to a more stable, manageable continuous pain was consistent with observations from other clinical reports of trigeminal neuralgia treated with cannabinoids [17, 20].

These findings are consistent with a large prospective Canadian study including more than 2000 patients authorized to use medical cannabis, of whom 68.8% had chronic pain as the primary condition. In that study, participants demonstrated statistically significant improvements in WHOQOL measures at follow‐up, particularly in physical and psychological domains, alongside reductions in pain severity and decreased opioid consumption [32]. Such data help contextualize the present observations within broader clinical experience.

The use of objective tools such as QualST and stereophotogrammetry also proved valuable in follow‐up. QualST enabled identification and monitoring of sensory changes, while stereophotogrammetry provided a three‐dimensional method to map areas of pain and allodynia. This quantitative documentation strengthens the correlation between subjective complaints and clinical findings. Previous studies using similar sensory assessment methods have underscored the relevance of combining subjective and objective measures in neuropathic pain research [5, 33].

Despite these observations, limitations inherent to case reports must be acknowledged. The absence of a control group and the small sample size preclude generalization, and individual responses to cannabinoids may vary according to genetic, metabolic, and psychological factors. An additional limitation is that both patients continued conventional pharmacological therapy during the observation period. Although medication regimens remained stable, the potential influence of concomitant treatments cannot be excluded and should be considered when interpreting outcomes. Therefore, the findings must be interpreted with caution. The observed changes do not allow causal or mechanistic inferences regarding cannabinoid therapy but should be regarded as hypothesis‐generating. The improvements observed may reflect individual variability, placebo effects, or interactions with concomitant therapies, reinforcing the need for controlled and adequately powered studies to clarify therapeutic mechanisms and clinical effectiveness. Additionally, the discontinuation phase and posttreatment follow‐up were relatively short, limiting conclusions regarding the durability of therapeutic effects and the potential occurrence of rebound symptoms after dose reduction or cessation. Longer follow‐up periods are necessary to determine the stability of analgesic response and long‐term safety in TNP.

Both patients reported meaningful functional improvements following cannabinoid therapy. One patient described regaining the ability to chew and brush her teeth without triggering shock‐like pain episodes. The other reported improved jaw movement and greater comfort during daily activities such as eating and oral hygiene. Both patients considered the treatment tolerable and perceived an overall improvement in their condition. The two clinical cases further reinforce the endocannabinoid system as a promising therapeutic target due to its role in regulating multiple physiological and pathological processes [34].

In summary, the present cases illustrate that adjunctive cannabinoid therapy was associated with reductions in pain intensity, changes in pain characteristics, and functional and psychosocial improvements in patients with refractory TNP. While consistent with existing literature, these observations remain preliminary and should not be interpreted as evidence of efficacy. Well‐designed controlled studies are necessary to determine safety, effectiveness, optimal dosing strategies, and the therapeutic role of cannabinoids in this clinical context.

## 4. Conclusion

This report describes two cases of refractory TNP in which adjunctive cannabinoid therapy was associated with improvements in pain characteristics, functional outcomes, and perceived quality of life, without serious adverse effects during the observation period. Given the inherent limitations of case reports, including the small sample and absence of a control condition, these findings should be interpreted with caution. Nevertheless, the cases highlight the potential clinical relevance of cannabinoids and support the need for well‐designed controlled studies to further investigate their safety, efficacy, and therapeutic role in the management of TNP.

## Author Contributions

Alex Moreira Mélo contributed to the conceptualization of the study, clinical investigation, data curation, and writing of the original manuscript draft. Luiz Guilherme Spadon‐Brito contributed to data collection. Júia Carrer Hallak, Glauce Crivelaro do Nascimento Marangoni, and Melissa de Oliveira Melchior contributed to manuscript review. Simone Cecílio Hallak Regalo and Jardel Francisco Mazzi‐Chaves contributed to data analysis and critical revision of the manuscript. Laís Valencise Magri supervised the study and contributed to conceptualization, validation, and critical revision of the manuscript.

## Funding

This study was supported by the Coordination for the Improvement of Higher Education Personnel (CAPES), Brazil.

## Disclosure

All authors read and approved the final version of the manuscript. The funding body had no role in the design of the study, data collection, analysis, interpretation of the results, or in writing the manuscript. All content was carefully reviewed and validated by the authors. The company had no role in the study design, data collection, analysis, interpretation of the results, or preparation of the manuscript.

## Ethics Statement

This study was approved by the Research Ethics Committee of the School of Dentistry of Ribeirão Preto, University of São Paulo (FORP/USP), under protocol CAAE: 92507925.7.0000.5419. All procedures were conducted in accordance with the principles of the Declaration of Helsinki.

## Consent

Written informed consent was obtained from both patients for the publication of this case report and the accompanying clinical images.

## Conflicts of Interest

The authors declare no conflicts of interest.

## Supporting Information

Additional supporting information can be found online in the Supporting Information section.

## Supporting information


**Supporting Information** The Supporting Information includes the CARE checklist (2013 version), an internationally recognized reporting guideline designed to improve the completeness, transparency, and methodological quality of case reports. The checklist comprises key items related to patient information, clinical findings, diagnostic assessment, therapeutic interventions, follow‐up, and outcomes. In the present study, the CARE checklist was used as a reference to guide the organization and reporting of clinical information, ensuring consistency with established standards for case report publication.

## Data Availability

The data that support the findings of this study are available from the corresponding author upon reasonable request.
