# Supplementary material for: Cannabinoid Therapy for Refractory Trigeminal Neuropathic Pain: Quantification of Somatosensory Alterations (QualST) by 3D Stereophotogrammetry—Two Case Reports
Source: Case Rep Dent. 2026 May 4;2026:7752444. doi: 10.1155/crid/7752444 (PMC13137124; doi:10.1155/crid/7752444)
Supplement: Supplementary file 1 — Supporting Information The Supporting Information includes the CARE checklist (2013 version), an internationally recognized reporting guideline designed to improve the completeness, transparency, and methodological quality of case reports. The checklist comprises key items related to patient information, clinical findings, diagnostic assessment, therapeutic interventions, follow‐up, and outcomes. In the present study, the CARE checklist was used as a reference to guide the organization and reporting of clinical information, ensuring consistency with established standards for case report publication. [file CRID-2026-7752444-s001.pdf]

CARE case report guidelines

CARE Checklist of information to include when writing a case report

(cc) BY-NC-ND

Topic	Item	Checklist item description	Reported on Line
Title	1	The diagnosis or intervention of primary focus followed by the words "case report"	1
Key Words	2	2 to 5 key words that identify diagnoses or interventions in this case report, including "case report"	11
Abstract (no references)	3a	Introduction: What is unique about this case and what does it add to the scientific literature?	8-9
	3b	Main symptoms and/or important clinical findings	9
	3c	The main diagnoses, therapeutic interventions, and outcomes	9
	3d	Conclusion—What is the main "take-away" lesson(s) from this case?	10
Introduction	4	One or two paragraphs summarizing why this case is unique (**may include references**)	12-20
Patient Information	5a	De-identified patient specific information.	48,58
	5b	Primary concerns and symptoms of the patient.	49,58,59
	5c	Medical, family, and psycho-social history including relevant genetic information	48,58
	5d	Relevant past interventions with outcomes	51
Clinical Findings	6	Describe significant physical examination (PE) and important clinical findings.	33-45, 49, 59
Timeline	7	Historical and current information from this episode of care organized as a timeline	24-25
Diagnostic Assessment	8a	Diagnostic testing (such as PE, laboratory testing, imaging, surveys).	33-45, 49, 59
	8b	Diagnostic challenges (such as access to testing, financial, or cultural)	not applicable
	8c	Diagnosis (including other diagnoses considered)	50, 60
	8d	Prognosis (such as staging in oncology) where applicable	not applicable
Therapeutic Intervention	9a	Types of therapeutic intervention (such as pharmacologic, surgical, preventive, self-care)	27-31, 52, 61
	9b	Administration of therapeutic intervention (such as dosage, strength, duration)	28-30, 36-37
	9c	Changes in therapeutic intervention (with rationale)	36-37, 55, 65
Follow-up and Outcomes	10a	Clinician and patient-assessed outcomes (if available)	53-55, 62-65
	10b	Important follow-up diagnostic and other test results	53-55, 62-63
	10c	Intervention adherence and tolerability (How was this assessed?)	52, 61, 63
	10d	Adverse and unanticipated events	52,63
Discussion	11a	A scientific discussion of the strengths AND limitations associated with this case report	68-82
	11b	Discussion of the relevant medical literature **with references**.	69-78
	11c	The scientific rationale for any conclusions (including assessment of possible causes)	71-76
	11d	The primary "take-away" lessons of this case report (without references) in a one paragraph conclusion	84-85
Patient Perspective	12	The patient should share their perspective in one to two paragraphs on the treatment(s) they received	81
Informed Consent	13	Did the patient give informed consent? Please provide if requested	Yes ☒ No ☐
